# Promoting Intersectoral Collaboration Through the Evaluations of Public Health Interventions: Insights From Key Informants in 6 European Countries

**DOI:** 10.34172/ijhpm.2020.19

**Published:** 2020-02-26

**Authors:** Sabrina Kriegner, Trygve Ottersen, John-Arne Røttingen, Unni Gopinathan

**Affiliations:** ^1^Oslo Group on Global Health Policy, Department of Community Medicine and Global Health and Centre for Global Health, Institute of Health and Society, Faculty of Medicine, University of Oslo, Oslo, Norway.; ^2^Division for Health Services, Norwegian Institute of Public Health, Oslo, Norway.; ^3^Research Council of Norway, Oslo, Norway

**Keywords:** Strategies for Evaluation, Promoting Intersectoral Collaboration, Public Health Interventions

## Abstract

**Background:** Intersectoral collaboration is critical to the successful implementation of many public health interventions (PHIs). Little attention has been paid to whether and how processes at the stage of evaluation can promote intersectoral collaboration. The objective of this study was to examine European experiences and views on whether and how the evaluation of PHIs promote intersectoral collaboration.

**Methods:** A qualitative study design was used. We conducted semi-structured interviews with 15 individuals centrally involved in the evaluation of PHIs in 6 European countries (Austria, Denmark, England, Germany, Norway, and Switzerland). Questions pertained to current processes for evaluating PHIs in the country and current and potential strategies for promoting intersectoral collaboration. Transcripts were analyzed using thematic analysis to identify key themes responding to our primary objective.

**Results:** Experiences with promoting intersectoral collaboration through the evaluation of PHIs could be summarized in 4 themes: (1) Early involvement of non-health sectors in the evaluative process and inclusion of non-health benefits can promote intersectoral collaboration, but should be combined with greater influence of these sectors in shaping PHIs; (2) Harmonization of methodological approaches may enable comparison of results and facilitate intersectoral collaboration, but should not be an overriding goal; (3) Involvement in health impact assessments (HIAs) can promote intersectoral collaboration, but needs to be incentivized and be conducted without putting overwhelming demands on non-health sectors; (4) A designated body for evaluating PHIs may promote intersectoral collaboration, but its design needs to take account of realities of policy-making.

**Conclusion:** The full potential for promoting intersectoral collaboration through the evaluation of PHIs appears currently unrealized in the settings we studied. To further promote intersectoral collaboration, evaluators and decision-makers may consider the full range of strategies characterized in this study. This may be most effective if the strategies are deployed so that they reinforce each other, value outcomes beyond health, and are tailored to maximize political priority for PHIs across sectors.

## Background

Public health interventions (PHIs), for the purpose of this study defined as population-based, preventive interventions, experience particular challenges with respect to receiving political priority and support for implementation compared to curative clinical services. Frequently cited reasons for lack of priority and support are that the benefits tend to be accrued over the long-term, are not easily visible, and are not immediately felt by individual beneficiaries.^1-5^ Moreover, a factor making implementation of PHIs particularly challenging is that these interventions in many instances require collaboration between sectors and action outside the health sector.^6,7^ This is partly because many of the factors targeted by PHIs are significantly shaped by decisions within the purview of sectors beyond the health sector.^8-11^ Accordingly, successfully increasing the priority for and implementing PHIs require ways of facilitating intersectoral collaboration.

Discussions on intersectoral collaboration in the context of PHIs have typically focused on how to facilitate intersectoral collaboration once it has been decided to implement a given PHI or in the context of influencing other sector’s policies and programs as part of implementing the Health in All Policies (HiAP) approach.^12,13^ Policy goals that imply shared responsibilities between sectors might also motivate intersectoral public health collaboration. Among the most prominent examples are the Sustainable Development Goals (SDGs).^8,9,14^

However, it might be desirable to facilitate intersectoral involvement at a much earlier stage. A promising strategy for facilitating intersectoral collaboration can be the evaluation of PHIs, since both the planning of an evaluation and the evaluative process holds many opportunities for bringing together stakeholders that have specific roles in the implementation of a PHI and whose interests are affected by the implementation.^15-22^ Evaluations can be designed to be deliberative processes where stakeholders across sectors weigh and balance different interests, negotiate what the evaluation should focus on and reach agreement about outcomes to measure. Of particular importance is using the opportunity to identify non-health benefits that can be valued by sectors outside the health sector.

Yet in spite of increased impetus for promoting intersectoral collaboration, particularly in wake of the SDGs, how evaluations of PHIs can be a tool for promoting intersectoral collaboration have, to our knowledge, received limited attention.^14,23^ From the literature (see ‘Methods’ for how these strategies were identified) several strategies for promoting intersectoral collaboration through the evaluation of PHIs can be identified (a definition and chief rationale of the strategies can be found in the main findings section).^6,21,24,25^ We currently know little about the potential of these strategies for promoting intersectoral collaboration through the evaluation of PHIs, which would be useful information for all actors seeking to improve the priority setting and implementation of PHIs. We also know little about the extent to which these strategies are actually utilized today. To fill this knowledge gap, we conducted a qualitative study focused on the experience with evaluating PHIs in selected European countries. The European context was selected because of the vast experience that has been accumulated with implementing various forms of intersectoral collaborations for health (eg, HiAP, healthy cities).^26,27^ Accordingly, government units responsible for evaluating PHIs might have gained experience with facilitating intersectoral involvement as part of the evaluative processes. The primary objective was to examine how the evaluation of PHIs best can promote intersectoral collaboration. We conducted a literature review to identify specific strategies for promoting intersectoral collaboration through the evaluation of PHIs, and explored the current experience with implementing strategies by interviewing individuals centrally involved in the evaluation of PHIs in selected European countries.

## Methods

### Theoretical Frameworks and Definitions

A PHI is commonly understood as an intervention whose primary objective is to promote health or prevent illness and that targets the entire or sub-groups of a population.^28,29^ We therefore defined PHIs as ‘population-based, preventive interventions’ for the purpose of this study. Interviewees were informed about this definition prior to beginning the interview. Under this definition, examples of interventions include those that aim to detect health risks in a population (eg, infectious disease surveillance), limit exposure to health risks (eg, plain packaging of tobacco), discourage unhealthy behaviors (eg, tax on sugar-sweetened beverages), promote healthy options in the entire population (eg, information campaigns) or a sub-population (eg, free fruits and vegetables in schools), change the underlying social and environmental conditions of risk for the entire population (eg, food reformulation), and facilitate healthier behaviors by changing individuals social or environmental context (eg, building bicycle lanes).^30-34^ In many cases, these types of PHIs have outcomes also beyond health and intersectoral collaboration is required for implementation. For example, free fruits and vegetables in schools require cooperation with the educational sector and can improve learning outcomes, while a tax on sugar-sweetened beverages requires adjusting fiscal policy and can have impact on commercial interests. The emphasis of our study was on these types of PHIs over PHIs that require much less consideration of the needs of other sectors (eg, vaccination programs). Our definition excludes clinical interventions that can also be preventive, but normally target individuals (eg, advice about smoking cessation to primary care patients).^28^

A literature review was conducted to identify specific strategies for promoting intersectoral collaboration through the evaluation of PHIs.^35-40^ These frameworks were used to inform the interview guide and to help interpret the qualitative findings. The search strategy was non-systematic and focused on literature about evaluations of PHIs, intersectoral collaboration in the context of PHIs, and options for promoting intersectoral collaboration. The primary search engines were Google Scholar and the electronic library databases of the University of Oslo and the University of Innsbruck. We chose Google Scholar because it also includes grey literature, which was a valuable source of information for this research purpose. Keywords used were “evaluation of public health interventions,” “intersectoral collaboration, evaluation, PHIs,” “across-sectors, evaluating PHIs,” or “actors, evaluation, PHIs.” Specific strategies for promoting intersectoral collaboration were delineated and defined after agreement between 3 of the authors (SK, TO, and UG).

### Study Design

A qualitative study design with semi-structured interviews as the primary data source was adopted for this study. Semi-structured interviews aimed to elicit views and experiences of individuals involved in the evaluation of PHIs.^41,42^ An interview guide was developed (Supplementary file 1), informed by a literature review of strategies for promoting intersectoral collaboration through the evaluation of PHIs.^41,42^ When designing the guide we also used Bryman’s checklist for interviews, which offers guidance on how to ensure the questions address the research issue and allow the interviewee to reflect on them from their perspective.^41^ Questions focused on 3 themes: (1) the current processes for evaluating PHIs; (2) the relationship between these evaluative processes and intersectoral collaboration; and (3) current and potential future strategies for promoting intersectoral collaboration through the evaluation of PHIs. Initial questions were open-ended, while subsequent questions asked respondents about 5 specific strategies highlighted in the literature (Table 1). Respondents were also asked to rank those 5 strategies in terms of importance for promoting intersectoral collaboration. Supplementary questions pertained to the appropriate role of cost-effectiveness in evaluating PHIs and how the evaluation of PHIs can best support priority setting among these interventions and clinical interventions. The interview guide was piloted in 2 interviews prior to those included in this study. The pilot-interviewees were both working for public health institutions in Austria (one in a public institute for health technology assessments and one in a private company for health and hospital). The interview guide was subsequently revised based on the suggestions from these pilot interviewees.

**Table 1:** Overview of Definition, Chief Rational and Main Themes Emerging From Key Informant Interviews on the Strategies

**Strategy the Theme Relates to**	**Definition**	**Chief Rationale**	**Main Theme Emerging From Interviews With Key Informants**
Non-health actors	The active and systematic involvement of actors from outside the health sector in the evaluation of PHIs^47^	Active and routine involvement of non-health actors promotes intersectoral collaboration by making these more aware of the benefits of PHIs pertaining to their sector and increase their sense of ownership with respect to these interventions^47,48^	Early involvement of non-health sectors in the evaluative process and inclusion of non-health benefits can promote intersectoral collaboration, but should be combined with greater influence of these sectors in shaping PHIs
Non-health benefits	The explicit and systematic inclusion of relevant non-health benefits in the evaluation^6,7^	Demonstration of non-health benefits increases the interest in funding and implementing many PHIs among actors outside the health sector^13^	
Harmonized methods	The harmonization of the methods used to evaluate interventions in different sectors^22,49^	Consistent evaluation of interventions across sectors makes it easier for the many actors to rally around the same interventions^22,49^	Harmonization of methodological approaches may enable comparison of results and facilitate intersectoral collaboration, but should not be an overriding goal
HIA	Encouraging actors outside the health sector with the task of considering health outcomes when evaluating interventions^50^	Consideration of health outcomes by non-health actors helps identify areas of common interest with health actors and can spur collaboration^47,51^	Involvement in HIAs can promote intersectoral collaboration, but needs to be incentivized and be conducted without putting overwhelming demands on non-health sectors
Designated body	The establishment of a designated national body for evaluating PHIs^52^	A designated body increases the visibility and the attention paid to PHIs both within and outside the health sector and enables better coordination among different actors^52,53^	A designated body for evaluating PHIs could bolster implementation of available strategies for promoting intersectoral collaboration but to be effective its design needs to take account of realities of policy-making

Abbreviations: HIA, health impact assessment; PHIs, public health interventions.

### Study Sampling

Purposive sampling was used to recruit interviewees for this study.^43,44^ We aimed to recruit interviewees who collectively could provide a breadth of experience and perspectives on the use of evaluations of PHIs to promote intersectoral collaboration. Purposive sampling occurred at 2 levels: country-level and individual level. At the country level, 6 countries were selected: Austria, Denmark, England, Germany, Norway, and Switzerland. These were selected based on each country having an identifiable government unit that is active in evaluating PHIs, and had experienced recent changes in the approach to evaluating PHIs.

Within each country, we purposively recruited individuals centrally involved in the evaluation of PHIs based on their employment or published research. In each of these countries, we reached out to institutions involved with evaluating PHIs and asked them to nominate interviewees. We also reviewed published literature on PHIs in the chosen countries and contacted authors of relevant publications. In addition, we asked European institutions in the area of public health and national health agencies to recommend potential interviewees (Supplementary file 2). For each country, we wanted to include interviewees from government and non-government agencies involved with the evaluation of PHIs, in order to obtain a broad range of perspectives.

Further candidates were identified by snowball sampling, ie, people contacted in the first round were asked to recommend other interviewees with relevant experience. Participants were contacted per email and repeated efforts were made, including phone calls. A total of 45 interviewees were invited to participate in the study and 15 interviewees accepted the invitation. The main reasons for declining were lack of relevant qualifications or expertise, or lack of time. All interviews were conducted by one researcher (SK) from the end of March until the beginning of May 2016. Six interviews were conducted by phone, eight via Skype, and one in person. Six interviews were conducted in German and nine in English. Table 2 summarizes the main characteristics of the respondents.

**Table 2:** Respondent Characteristic

**Countries**	**N**	**Gender**		**Type of Employer**		
		**Male**	**Female**	**University**	**Government Unit**	**Private Company**
Austria	4	3	1	2	2	-
Denmark	2	-	2	1	1	-
England	2	-	2	1	1	-
Germany	3	2	1	1	1	1
Norway	2	1	1	1	1	-
Switzerland	2	2	-	-	1	1
Total	15	8	7	6	7	2

There were 2 respondents from each country except Austria (4) and Germany (3) (Table 2). Seven were government employees and 8 were employed outside government. Interviewees employed outside government came from universities (6) and private companies (2).

Participants employed by universities included lecturers, adjunct professors, researchers, post-doctorates and head of departments. Interviewees from national or regional governmental public health institutions worked as executive or deputy directors, head of departments or senior advisors. Participants representing private companies served in the role as executive director or head of department in for-profit organizations in health evaluation and research.

Participants got the option to choose between German and English as the language for the interview and correspondence. Informed consent was obtained prior to each interview to audio record the conversation.

### Data Analysis

The interviews were transcribed non-verbatim, correcting grammatical errors, and removing filler words, false starts, stutters, and sounds insofar these corrections did not influence the meaning of what was expressed. The transcripts were kept in the original language as long as possible and interviewees were asked how to best translate certain statements to English during the interviews or via email correspondence afterwards if necessary.

Two analytical strategies were implemented. The first followed the principles of the template organizing style,^45,46^ whereupon the 5 strategies identified in Table 1 were used as pre-existing categories. Accordingly, interviews were analyzed for insights about the use of these strategies and their potential for promoting intersectoral collaboration during the evaluation of PHIs. The second analytical strategy entailed an inductive approach to identify main themes raised by the interviewees, broadly following the process described by Yin.^42^ A 3-staged process to establish common patterns across all interviews was followed. During the first stage, the investigator focused on coding broader, individual statements of the interviewees relevant to the research questions (also known as “level 1 codes”). The second stage involved comparing the broader individual statements across interviews, and sought to summarize and combine level 1 codes under level 2 codes reflecting broader concepts capturing the meaning of the qualitative data. Finally, we identified overarching themes that covered one or more level 2 codes, and which generated insight about the 2 primary objectives: (1) the experience with implementing the strategies described in Table 1 and; (2) how the evaluation of PHIs best can promote intersectoral collaboration.

Transcripts were coded by one investigator (SK). Each theme and the codes associated with these themes were reviewed by and discussed with the co-authors (UG and TO). A table of qualitative codes can be found in the supplementary materials (see Supplementary file 3).

## Results

The literature review identified specific strategies for promoting intersectoral collaboration through the evaluation of PHIs. A definition and chief rationale for each of these strategies is summarized in Table 1, along with the main themes emerging from interviews with key informants on the current experience with the implementation of these strategies.

### Early involvement of non-health sectors in the evaluative process and inclusion of non-health benefits can promote intersectoral collaboration, but should be combined with greater influence of these sectors in shaping PHIs

Intersectoral involvement from sectors such as education, housing transport, welfare and social assistance, and labor is integral to many PHIs; yet motivating the involvement of these and other sectors for the support of PHIs, and intersectoral action for health more generally, have proven challenging.^13,54^ Theories and research from public administration and political science about how to transcend sectoral boundaries and engage with and motivate the involvement of other sectors have, among other factors, identified inclusive participation of relevant actors and broadening the epistemic community during knowledge creation and utilization as factors that can promote better integration.^54,55^ We identified interviewees to make the similar case with respect to how evaluations of PHIs could promote intersectoral collaboration. Interviewees highlighted the importance of a participatory evaluation approach and the need to involve all institutions that play a role in the intervention in order to motivate their engagement with PHIs. It was emphasized that the deliberative process of an evaluation could pinpoint where action across sectors would be necessary to effectively implement PHIs.

Of particular importance was to involve non-health actors from the very beginning of the evaluation process in order to foster their inclusion, and for their involvement to be effective and sustainable. Interviewees stressed that the *non-health benefit* and *non-health actor* strategies are strongly connected, arguing that involvement of actors outside the health sector automatically would lead to increased consideration of non-health benefits. An emphasis was placed on making non-health benefits measurable and visible during the evaluative process, and to include “*intermediate outcomes that are not directly related to health and relate them to health through research*” (Interviewee 10, Norway). Moreover, inclusion of non-health benefits in the evaluation of PHIs could facilitate the involvement of experts from outside the health sector and help demonstrate how a PHI benefits these sectors. The non-health benefits strategy could be a starting point for detecting where collaboration would be useful. For example, one interviewee expressed:

*“Including other aspects in the evaluation is really important. I am not sure if this is able to create new collaborations, but it could be a start, like an initialization to do so*” (Interviewee 14, Switzerland).

Implementation of HiAP approaches are among the most prominent examples of systematic efforts to promote cross-sectoral activity. Experience with using evaluations actively to promote intersectoral collaboration is, however, sparse in the literature. One example is the experience with health lens analysis, which was implemented as part of HiAP in South Australia.^12^ Although primarily applied to examine policy issues and actions outside the health sector, health lens analysis includes features that evaluations of PHIs more generally could benefit from: (1) there is a systematic examination of connections among policy, strategies and health; (2) there is an emphasis on broad involvement early in the process; (3) the focus is not only on improving health, but also identifying non-health benefits.^56^ This involvement seemed to have motivated public servants across departments in South Australia to appreciate the impact of their work on public health, and claim a greater stake in maximizing positive health impact.^56^

In our study, however, a few interviewees noted that including non-health actors in PHI evaluations would alone be insufficient to motivate greater engagement in PHIs. One interviewee expressed:

“*What then is the motivation for them to get involved if they can’t have any say or influence on what we decide to do*” (Interviewee 12, England).

Accordingly, it was argued that the involvement of non-health actors and the consideration of non-health benefits should be complemented by these actors being allowed greater room to shape PHIs. Being invited to evaluate PHIs after it has been decided to implement them was considered insufficient; motivating involvement also require creating space for the voice of relevant sectors in the public health policy-making process. This particular point emphasizes the ownership of PHIs and the goals these interventions are designed to pursue.^54^ A particularly challenging issue is when the intervention demands buy-in from other sectors, but where the ownership and policy-making process solely rest in the health sector.^54,57^

### Harmonization of methodological approaches may enable comparison of results and facilitate intersectoral collaboration, but should not be an overriding goal

Views differed about the value of harmonizing methods for evaluating PHIs. Many interviewees interpreted this as the use a single methodology for all sectors. Three interviewees expressed concerns that harmonization (understood as a “one size fits all approach”) would require a lot of work and questioned the value of such an effort. One interviewee argued that sectors document and evaluate interventions with different methods, and that differences existed for justified reasons:

“*Evaluation is very heterogeneous and this also has its reason or its cause. Interventions are very different; you also need different methods for these interventions to really be able to evaluate them and answer the questions which need to be answered*” (Interviewee 14, Switzerland).

However, interviewees believed that harmonization in terms of sharing guidelines, indicators or outcomes would facilitate consistency between evaluations. This kind of harmonization could facilitate comparison of results from evaluations and thus promote intersectoral collaboration. Overall, these experiences and views were consistent with the literature stating that aligning methods should not result in applying one methodological tool to every intervention related to health.^49,58^ Instead, what could be useful is establishing certain guidelines or criteria to fulfill when evaluating interventions in specific areas, which should make it easier to compare the outcomes and effectiveness of interventions.^49,58^

### Involvement in health impact assessments can promote intersectoral collaboration, but needs to be incentivized and be conducted without putting overwhelming demands on non-health sectors

Almost all interviewees (n = 12) believed that health impact assessments (HIAs) can promote intersectoral collaboration. HIA is a systematic approach involving different procedures, methods and tools to assess the potential effects of a policy, program or project on the health of a population.^59^ Interviewees experienced that such a process can promote joint understanding across various sectors with respect to public health policy. To succeed with this strategy, several points were described. HIAs need to be simple and easy to apply, and those conducting HIAs also need to see the benefits of doing this. For example, one respondent stated that:

*“We need to do it in a very non-bureaucratic way and in a way that people don’t feel that is something we also have to do, but something they can see an interest in”* (Interviewee 13, Denmark).

However, interviewees also expressed reservations about this strategy. One interviewee questioned the need to establish the connection between health and non-health issues all the time, arguing that the many links are well-known. Moreover, 2 other interviewees expressed that there were risks associated with a too strong emphasis on health outcomes when seeking to collaborate with actors outside the health sector. Non-health actors have their own primary objectives and may become overwhelmed by a demand to also prioritize health. It was therefore deemed crucial that public health policy objectives and associated HIAs are not perceived as an additional burden by non-health sectors, consistent with literature indicating that seeing health as an additional task is a key challenge to achieving support for an intersectoral health agenda.^60,61^ Accordingly, interviewees expressed the need for humility when engaging with other sectors. The concerns raised by these interviewees are consistent with what is referred to as “health imperialism”—understood as defining a collaborative agenda from a health perspective alone, and only considering how other sectors can contribute to the goals of the health sector without recognizing the interests of other sectors.^62^ An alternative approach would be to explore what actions other sectors already have in place or could begin with to support a common health agenda, and prioritize goals that entail benefits to all the sectors involved.^63^ Furthermore, the health sector can engage in a ‘cooperation strategy’ where the health sector does not necessarily stress its own objectives, but rather aims to advance public health by providing its own expertise to other sectors to assess if it can contribute to their policy goals.^63^ Experiences from implementing HiAP in for example South Australia and Finland suggest that incentivizing collaboration from non-health sectors in these ways are more promising strategies.

### A designated body for evaluating PHIs could bolster implementation of available strategies for promoting intersectoral collaboration but to be effective its design needs to take account of realities of policy-making

A crucial question raised by the previous themes is whether intersectoral collaboration in the context of PHIs can be strengthened beyond processes specific to a single evaluation. Interviewees supported the idea that a designated body for evaluating PHIs at the national level could be promising for intersectoral collaboration. Public Health England and the Federal Office of Public Health in Switzerland were pointed to as examples of such bodies, while in Norway efforts were under way to establish a designated competence center for the evaluation of PHIs. It was raised that a designated body could play multiple roles to facilitate intersectoral collaboration. First, it was argued that such a body could mobilize additional resources and attention to the evaluation of PHIs. Second, it could actively use evaluations of PHIs to bring key actors together and facilitate cooperation. Finally, a centralized entity could be well-positioned to ensure dissemination of PHI evaluations to a broad range of actors, and use the learning to implement the most promising interventions.

While a designated body was considered a promising strategy, interviewees argued that the advantages of working across sectors to address the broader health benefits need wide acceptance among politicians for evidence to be reflected in their decision-making. A wider interpretation of this point is that the design of a designated body for evaluating PHIs needs to take account of the realities of the policy-making process.^64-66^ Among the key design features that interviewees emphasized were a clearly defined mandate, and membership with a multi-sectoral and multi-disciplinary background. With respect to intersectoral collaboration, these 2 features speak to insights generated from theories and empirical research from political science and public administration that indicate the value of securing a designated body authority and ability to convene inclusive processes that engage across sectors.^64,66^ For example, the experience with implementing HiAP in South Australia indicates that a HiAP unit with a political mandate to undertake Health Lens Analysis was crucial to making health a legitimate concern among other sectors.^12^

## Discussion

The importance of non-health sectors for improving public health have long been recognized, but full-fledged appreciation and corresponding action are still pending.^23,61,67-73^ Meanwhile, the critical role of intersectoral collaboration is becoming ever more evident, with a major case in point being the SDGs.^74^ These 17 goals and 169 targets represent a universal and comprehensive agenda, exposing how different social objectives and different sectors are closely intertwined. Accordingly, the SDGs represent a unique framework to promote and structure collaboration across diverse actors^75,76^ and a statement that progress mandates intersectoral collaboration. Our study, considered together with relevant literature, indicates that evaluations of PHIs can be used more actively by policy-makers and social planners as a tool for promoting intersectoral collaboration. Experiences from the interviewees indicated considerable potential in multiple strategies for using evaluations more actively. However, 3 aspects merit careful consideration if the full potential of strategies highlighted by this study are to be fulfilled: (1) Synergies; (2) the importance of political support and; (3) the dangers of overemphasizing public health as an intersectoral policy goal (eg, ‘health imperialism’). We consider each of these in the subsequent sections.

### Synergies

For policy-makers, social planners, and researchers it is worth considering synergies among the available strategies for promoting intersectoral collaboration. Our findings suggest that the non-health actor and non-health benefit strategies can very well be integrated, since the involvement of actors outside the health sector in the evaluation is likely to facilitate the consideration of non-health benefits. The involvement of such actors and the inclusion of such benefits may also facilitate the harmonization of methods across sectors and the use of HIA. The link between the inclusion of non-health benefits in evaluations within the health sector and the use of HIAs outside the health sector pertains to the issue of symmetry. If actors in non-health sectors are expected to consider health outcomes, actors in the health sector could be expected to consider important non-health outcomes, such as those related to education, to the economy, and to the environment. A designated body for the evaluation of PHIs could help promote all these other strategies and do so in an integrated way.^54^ Empirical studies of whether designated bodies for evaluating PHIs have successfully promoted intersectoral activity is sparse in the literature, but a few examples exist. In Finland, 2 examples underscore the potential benefits of centralized guidance for PHIs. On one hand, an Advisory Board for Public Health was established to serve as a coordinating mechanism for implementing Finland’s public health strategy known as Health 2015,^77^ which among other things reduced the burden of overstretched civil servants in monitoring and evaluating the progress of implementation.^78^ On the other hand, dismantling the state-level steering mechanism lead to lack of an entity to produce normative guidance, and to direct and monitor the municipalities work on public health and health promotion.^77^ Another example is from Sweden, where Lundgren describes how the Swedish National Institute of Public Health, in charge of coordinating and evaluating public health, facilitated opportunities to bring together all important stakeholders as well as disseminate knowledge and data about the health dimension of various sectoral agencies.^79^ Finally, during the implementation of HiAP in South Australia a dedicated HiAP unit in the Department of Health was responsible for Health Lens Analysis and was experienced to have a positive influence on the involvement of other sectors.^80^

### Dangers of Overemphasizing the Health Perspective (eg, “Health Imperialism”)

HIAs is a strategy that entails encouraging actors outside the health sector to explicitly integrate health outcomes in their evaluations.^50^ Such a strategy can be perceived as actors in the health sector pushing for higher priority to health vis-à-vis social objectives anchored in other sectors.^81^ A push for harmonization of methods for evaluation may also be interpreted in a similar way, especially if the methods currently employed in the health sector are favored. The dangers of such “health imperialism” was indeed stressed by some of the interviewees of this study, who called for actors in the health sector to be sufficiently humble when engaging with actors in other sectors and to go beyond a single-minded pursuit of objectives directly focused on health. This is in line with more general warnings not to deter implementation of intersectoral collaboration by solely defining and designing collaborations from a health perspective.^14,52,53,61-63,73,80,82^ Against this background, the strategies of including non-health actors and non-health benefits may be particularly welcome. These strategies can be seen as going in the opposite direction, ie, facilitating early and active involvement of non-health sectors in assessments of PHIs and ensuring that a broader range of social objectives are taken into account by actors in the health sector. It thus brings more symmetry into the evaluation of PHIs and a stronger basis for mutual understanding and collaboration across sectors.

### Priority and Political Support

Today, the evidence for PHIs is often considered less rigorous and comprehensive than for clinical services^83^ and has been charged for being too narrow to expose the full value of these interventions.^7,84^ In addition to non-health outcomes, evidence on the interventions’ distributional impact is in particular short supply. This is unfortunate given the aim of reducing inequalities in many countries and the major contributions PHIs can make to this end.^22,53,85,86^ Yet better evidence needs not automatically translate into higher priority or stronger political support for specific PHIs or public health more generally.^87-90^ Factors beyond evidence are often more important and may shape priorities much more directly.^91-93^ Accordingly, a precondition for creating a virtuous circle between political support and the strategies highlighted by this study is that their deployment take account of the realities of the public health policy-making process. For example, a designated body for evaluating and disseminating knowledge about PHIs would in addition to a strong political mandate for engaging across sectors, also need the ability to tailor knowledge translation depending on the PHI in question. Drawing on work by Fafard and Hoffman,^64^ a designated body for evaluating PHIs may pay attention to: (1) the broad range of people that might be involved in a public health policy decision and to whom the evidence must be communicated (audience make-up); (2) whether the policy issue is managed by specialized officials within the health sector or requires buy-in across government (audience breadth); (3) characteristics of the policy network, eg, the people and groups inside and outside of government that shape public health policy, as well as the broader policy advisory system beyond the specific designated body (policy context) and; (4) whether the PHI involves regulation, communication, taxation, or spending, or a combination of these, which influence the complexity of the policy-making process (policy instrument). By using the evidence-base from political sciences, public administration and policy studies that demonstrate the particular challenges with evidence use for public health policy, evaluative processes for PHIs can be better designed to promote intersectoral collaboration.

## Limitations

This study recruited interviewees from different European countries to enable collection of qualitative data that could shed light on the research questions from a broad range of experiences and perspectives. Countries may differ with respect to how the system for evaluating PHIs is organized, the political priority for public health and the extent to which whole-of-government approaches are institutionalized. These are highly influential factors affecting the institutional environment and the incentives to promote intersectoral collaboration. While this study included country experiences providing valuable insights, it was not designed to provide in-depth comparison of country experiences. A future study could be designed to stratify the sample by country and undertake in-depth case studies. We only interviewed respondents working within the health sector. The perspectives of actors in other sectors are obviously crucial to consider when designing initiatives to strengthen intersectoral collaboration for health, and future studies may include more respondents from outside the health sector.

## Conclusion

This study, informed by 15 interviewees working with evaluation of PHIs across 6 European countries, suggests that the full potential of promising strategies for promoting intersectoral collaboration through the evaluation of PHIs are currently unrealized. Effective use requires that these strategies are deployed so they reinforce each other, avoid solely overemphasizing the health perspective and are tailored to maximize political priority for PHIs across sectors.

## Acknowledgements

The authors thank all the interviewees for their participation, and the 4 peer-reviewers for providing valuable feedback.

## Ethical issues

The Data Protection Official of the Norwegian Centre for Research Data granted permission for this study (project no. 47911). Participants received verbal information about the project and gave consent to participe. Additionally, participants approved quotations prior to publication.

## Competing interests

From January 1, 2017, TO has served as executive director at the Norwegian Institute of Public Health. The Institute is involved with evaluation of PHIs and have been tasked with building up the competence centre for the evaluation PHIs.

## Availability of data and material

In line with the notification form from the Norwegian Social Science Data Services, data will be stored on a private computer and will be made anonymous by the end date of this project (31.01.2020).

## Authors’ contributions

SK led the design of the study, conducted the interviews and collected other data, prepared the first draft of the manuscript, and was responsible for consecutive revisions. TO and UG provided supervision throughout. TO, UG, and JAR provided input on the study protocol and drafts of the manuscript.

## Authors’ affiliations

^1^Oslo Group on Global Health Policy, Department of Community Medicine and Global Health and Centre for Global Health, Institute of Health and Society, Faculty of Medicine, University of Oslo, Oslo, Norway. ^2^Division for Health Services, Norwegian Institute of Public Health, Oslo, Norway. ^3^Research Council of Norway, Oslo, Norway.

## Supplementary files

Supplementary file 1. Interview guide.

Supplementary file 2. List of institutes that have been contacted for the interview study.

Supplementary file 3. Analysis and qualitative codes.

## Key Messages

**Implications for policy makers**

- There might be considerable potential in promoting intersectoral collaboration through the evaluation of public health interventions (PHIs), and the experiences collected across 6 European countries indicate that the evaluative phase can be used more actively as a tool for promoting intersectoral collaboration.
- Specific strategies for promoting intersectoral collaboration through the evaluative process include involving non-health actors actively in the process, including non-health benefits, harmonizing methods across different types of sectors, inclusive health impact assessments (HIAs), and establishing a designated body for evaluating PHIs that has the authority and ability to convene inclusive processes and deploy knowledge translation approaches tailored to the specific PHI in question.
- To further promote intersectoral collaboration, evaluators and decision-makers may consider the full range of strategies characterized in this study. This may be most effective if the strategies are deployed so that they reinforce each other, value outcomes beyond health, and are tailored to maximize political priority for PHIs across sectors.

**Implications for public**

Public health interventions (PHIs) are crucial for promoting health and reducing health inequalities, and the benefits of such interventions go far beyond the health sector. This study characterizes key strategies for promoting intersectoral collaboration in order to realize these benefits, including non-health actors, non-health benefits, harmonized methods, health impact assessments (HIAs) and designated body, and experiences applying them. The successful application of these strategies can give PHIs greater prominence in health policy-making.
