# Supplementary material for: Promoting Intersectoral Collaboration Through the Evaluations of Public Health Interventions: Insights From Key Informants in 6 European Countries
Source: Int J Health Policy Manag. 2020 Feb 26;10(2):67–76. doi: 10.34172/ijhpm.2020.19 (PMC7947666; doi:10.34172/ijhpm.2020.19)
Supplement: Supplementary file 1 — Interview guide. [file ijhpm-10-67-Supp1.pdf]

Supplementary file 1

Interview guide

Introduction

The purpose of the series of interviews of which this interview will be part is to gather information about.

1) current approaches to the evaluation of public health interventions (PHIs) in selected European countries;

2) how the current approaches promote intersectoral collaboration (IC); and

3) how these approaches can be revised to better promote such collaboration.

To this end, we will in this interview ask you about your personal views and experiences with regard to these matters.

With PHIs, we here mean population-based, preventive interventions. With IC, we here refer to collaboration among two or more sectors of government or society. One of these may be the health sector.

We hereby invite you to participate in an interview. You can opt out any time during the interview, without any other consequences than that your responses will not be included in the study. We ask for your permission to tape-record this interview. We plan to use your input in a master thesis and one or more journal articles, but we will not reveal your identity, and we will seek your permission before we quote you.

Do you agree to participate in this interview?

"Facesheet"-information

1. General kind: name
2. Specific kind: country of work, institution, position in institution, role of institution and respondent in evaluation of PHIs

Current situation

3. In broad terms, how do [the country in question] evaluate PHI today?

    3.1. What actors and institutions are involved in the process of evaluation and how? (Do you have a designated body for the evaluation of PHIs?)

3.2. What tools or methods are used, if any?

3.3. Which benefits are considered?

3.4. Is there any systematic link to the evaluation of clinical services?

3.5. How does this differ from the past? And are there any changes planned?

PHI and IC

5. Do you think the way PHIs are evaluated can affect IC? If so,

   5.1. What features of the evaluation of PHIs can promote or hinder IC in your view?

6. Are there any specific strategies promoting IC? How would the approach for evaluating PHIs need to be modified in order to promote IC?
7. How important is the evaluation of PHIs for promoting IC? (After initial response: how important is the evaluation of PHIs for promoting IC compared to other ways of promoting IC, such as HiAP (Health in All Policies) or cross-sectoral forums?)

Specific strategies promoting IC

We will now describe some possible features of the evaluation of PHIs and ask about your views on the role of these in promoting IC.

8. **Non-health benefits**: Do you think it can promote IC to evaluate benefits other than health improvements? (If asked what is meant by "benefits other than health improvements:" benefits that do not themselves represent changes in health, but these benefits may affect health or be affected by health status.)(If asked for examples: educational or financial benefits). If so, how?

   8.1. Which non-health benefits do you think are important to include in the evaluation of PHIs?

   8.2. If you could only involve one other kind of benefit than health improvements in the evaluation of PHIs, which benefit would that be?

9. **Non-health actors**: Do you think it can promote IC to include actors other than those in the health sector when evaluating PHI? If so, how? (If asked for examples: people or institutions in the education, transport, or social sector).

   9.1. If you could only involve one other sector than the health sector in the evaluation of

PHIs, what sector would that be?

9.2. Why do you think this sector is important to include in an evaluation of a PHIs?

10. **Designated body**: Do you think having a designated national body for the evaluation of PHIs can promote IC?

    10.1. If so, what would be important qualities of such a body?

    10.2. What would be needed to ensure the quality and legitimacy of such a body?

11. **Harmonized methods**: Do you think it can promote IC if actors in different sectors harmonized their methods for evaluating interventions with health impacts?

12. **Health impact assessment**: Health impact assessments examine the impact of non-health policies on the health of a population with the help of different qualitative and quantitative tools and procedures. Do you think health impact assessments can promote IC?

Ranking of strategies in terms of importance

13. Can you please rank the strategies we have discussed in this interview according to their importance for promoting IC? Please include in your ranking both the strategies you have suggested and the strategies we specifically asked you to consider.

Other questions

14. In general, what role do you think information on cost-effectiveness should play in the evaluation of PHIs?

15. In general, how do you think the evaluation of PHIs can be done to best help prioritize among these interventions and clinical interventions?

16. Is there anything you would like to add?

Definitions

PHIs: population-based, preventive health interventions.

IC: collaboration among two or more sectors of government or society. One of these may be the health sector.
