# Supplementary material for: Promoting Intersectoral Collaboration Through the Evaluations of Public Health Interventions: Insights From Key Informants in 6 European Countries
Source: Int J Health Policy Manag. 2020 Feb 26;10(2):67–76. doi: 10.34172/ijhpm.2020.19 (PMC7947666; doi:10.34172/ijhpm.2020.19)
Supplement: Supplementary file 2 — List of institutes that have been contacted for the interview study. [file ijhpm-10-67-Supp2.pdf]

Supplementary file 2

List of institutes that have been contacted for the interview study

Austrian Public Health Association

Berlin School of Public Health, Germany

Bundesamt für Gesundheit / Federal Office for Health, Switzerland

Bundesministerium für Gesundheit / Federal Ministry for Health, Germany

Bundeszentrale für gesundheitliche Aufklärung / Federal Center for health education

Department for evidence-based medicine, Donau-University Krems, Austria

Department Health and Health Service Research, Ludwig Boltzmann Institute for Health Technology Assessment

European public health association

Evidence based medicine and health technology assessments, Hauptverband der österreichischen Sozialversicherungsträger / Main association of the Austrian social insurance agencies

Forschung Beratung Evaluation GmbH (Gesellschaft mit beschränkter Haftung / limited liability company), Germany / Research Consultation Evaluation GmBH

Gesundheit Österreich GmBH / Health Austria GmBH

Health Promotion, University of Bergen, Norway

Ministry of health, Austria

National Institute for Health and Care Excellence, England

Norwegian Directorate of Health

Norwegian Institute of Public Health

Postgraduate Public Health Program, Medical University Graz, Austria

Public Health England

Public health, Unit for health promotion research, University of Southern Denmark

Public Health, University of Bremen, Germany

Public Policy and Health, Durham University, England

Qualität & Evaluation / Quality & Evaluation, Switzerland

Robert Koch Institute, Germany

UK Health Forum

University Bielefeld, Germany

University of Cambridge

University Vienna
